# Influence of the Tool Cutting Edge Helix Angle on the Surface Roughness after Finish Milling of Magnesium Alloys

**DOI:** 10.3390/ma15093184

**Published:** 2022-04-28

**Authors:** Ireneusz Zagórski, Anna Szczepaniak, Monika Kulisz, Jarosław Korpysa

**Affiliations:** 1 Department of Production Engineering, Mechanical Engineering Faculty, Lublin University of Technology, 20-618 Lublin, Poland; anna.szczepaniak1@pollub.edu.pl (A.S.); j.korpysa@pollub.pl (J.K.); 2 Department of Organisation of Enterprise, Management Faculty, Lublin University of Technology, 20-618 Lublin, Poland; m.kulisz@pollub.pl

**Keywords:** finishing milling, magnesium alloys, surface roughness, artificial neural networks, statistical analysis

## Abstract

This paper shows the surface quality results after finishing milling of AZ91D and AZ31 magnesium alloys. The study was performed for variable technological parameters: cutting speed, feed per tooth, axial depth of cut and radial depth of cut. The tools used in the study were two carbide cutters with a different tool cutting edge helix angle. The measurement of the research results presented the surface roughness parameters was made on the lateral faces and the end faces of the specimens. Statistical analysis and simulations using artificial neural networks were carried out with the Statistica software. The normality of the distribution was examined, and the hypotheses of the equality of mean values and variance were verified. For the AZ91D magnesium alloy on the lateral and the end faces (Ra, Rz parameters), simulations were carried out. Two types of ANN were used: MLP (Multi-layered perceptron) and RBF (Radial Basis Function). To increase the machining stability and to obtain a high surface finish, the more suitable tool for finishing milling is the tool with a helix angle of λ_s_ = 20°. Artificial neural networks have been shown to be a good tool for predicting surface roughness parameters of magnesium alloys after finishing milling.

## 1. Introduction

In finish machining, the standard and final requirement is the production of parts with a precise shape, specific dimensions and high-quality surface. The ultimate objective is to make milling the final machining operation, thus eliminating, e.g., the process of finish grinding that generates harmful grinding dust. The geometric structure created in this way is defined as the overall surface condition and is the final stage of the process of machining a given part.

The surface analysis is carried out by researchers around the world sometimes these studies concern analysis in terms of tribological properties and tribological wear [1] (through, inter alia, the application of protective layers [2]), or the analysis of the suitable tuning of the local composition and atomic arrangement to obtain excellent mechanical properties [3]. Another area of research related to surface analysis relates to the geometric structure of the surface, which consists of waviness, roughness, shape errors and structure directionality, whereas the surface quality includes the following, roughness, waviness and surface defects. It should be noted that the considered surface should be analysed, taking into account its functional and strength characteristics. Whereas the geometrical structure of the surface and the physical properties of the surface layer (including its structure, hardness and internal stress) constitute the so-called technical quality of the product [4,5].

One of the most frequently analysed and well-known surface roughness parameters (despite there being more than 30 of them) is the Ra parameter. Nevertheless, it does not provide information on the shape of the profile and individual peaks that only slightly affect the value of this parameter. Since the parameter is an absolute value, it does not include the valleys or peaks of the profile. The second most common parameter used in production workshops for checking surface quality is the highest roughness profile height—Rz. This parameter partially includes individual valleys or peaks, so it should be mainly analysed and taken into consideration in the case of bearing or sliding surfaces and measurement surfaces [4].

Together with the Rz parameter, the Rp and Rv parameters should be analysed. The Rp parameter provides information about the profile outline and the abrasion resistance of the tested surface. A surface with lower abrasion resistance is characterised by higher Rp values compared to the Rv values. In addition to the Ra parameter, the Rq parameter can be analysed due to its greater sensitivity to individual single valleys or peaks. However, it is impossible to analyse the distribution of peaks, and, as in the case of Ra, the parameters do not include information on whether the profile is characterised by peaks or valleys. Quite simplified, it can be assumed that for a profile of a random nature, this relationship can be defined as Rq ≈ 1.25 Ra [5].

The significant parameters that allow the analysis of the surface functionality are the roughness profile asymmetry coefficient Rsk and the roughness profile slope coefficient Rku [6]. These coefficients require a series of measurements, but they play an important role in monitoring the technological process, and they are useful for detecting surface defects. A surface with negative skewness is characterised by a greater frequency of deep valleys, defined as a plateau, and is considered optimal. The symmetric distribution of the profile is reflected by zero skewness. Rsk can be used to monitor conductivity, keep lubricants and support technological process analysis. Moreover, surfaces with a positive Rsk show good adhesion resistance. In the case of Rku, high values (above 3) indicate the presence of sharp peaks and grooves, while values below 3 indicate that the peaks and grooves are rounded. In conditions without the use of lubricant, it was shown that surfaces with a high Rku value and a positive Rsk value should result in a lower static friction coefficient compared to surfaces with Rku = 3, Rsk = 0 [7]. In addition to the traditional measurements of surface roughness, more and more often, there are works devoted to alternative methods of determining the surface structure, e.g., multi-scale methods [8,9]. These methods make it possible to analyse surfaces with even greater accuracy and detail. Their use allows for additional improvement of the quality of manufactured elements. Among these methods can be distinguished, among others, the Wavelet transformation, which enables the evaluation of the surface structure on the basis of the signals obtained during the measurements. Since the signals largely contain noise and interference, it is necessary to develop appropriate solutions to minimise them [10]. For example, in the work of Gogolewski et al., the images registered with the optical measuring instruments were decomposed using the two-dimensional wavelet transform to determine the minimum chip thickness in face milling [11]. However, multi-scale methods are quite a new solution; therefore, the use of commonly known surface roughness parameters compliant with the ISO standard allows for an easier understanding of the surface condition.

Both the surface roughness and the dimensional accuracy [12] are often important indicators in many manufacturing processes. It has been checked and proven that it is possible to achieve low values of roughness parameters (and thus a high quality final surface) after rough milling with cutters equipped with PCD inserts with Ra ≤ 0.16 µm [7]. Similarly, it has been observed that it is possible to obtain an IT2–IT5-level accuracy [12] with the use of high-quality carbide cutters with or without protective coatings. Similarly, when facing larger surfaces (e.g., the end face), it is possible to obtain the Ra parameter in the range of 0.2–0.8 µm for face milling cutters with PCD inserts. In this case, the Mg-Ca0.8 alloy was analysed [13]. Similar values of the Ra parameter (approx. 0.4 µm) were obtained with a combination of dry milling and low plasticity burnishing [14]. However, in subsequent research on the Mg-Ca0.8 alloy, [15] Ra at the level of approx. 0.9–1.4 μm in milling and Ra at the level of approx. 0.09–0.8 μm in inverse milling were obtained. In these tests, a face milling cutter with uncoated carbide inserts was used.

In other research works [16], the machinability of the Mg-Ca1.0 alloy was analysed. During milling, the Ra parameter obtained was at the level of approx. 0.08–0.16 μm. A face milling cutter with DLC coating (“diamond-like” coating) was used in the research tests. Whereas for milling the AM60 alloy, the Ra parameter obtained was at the level of approx. 0.3 µm [17]. Additionally, during the scientific research, the following was analysed: the geometry of the cutting tool edge and the presence or absence of protective coatings. In the analyses of different tool rake angles (γ = 5° and γ = 30°), the Ra obtained for γ = 5° was approx. 0.5 µm (γ = 5°, end face of specimen), while the Ra obtained for γ = 30° was approx. 2 µm (γ = 30°, end face of specimen). The value of the Ra parameter on the lateral face of the specimen was approx. 0.3 µm. The research was carried out for the rough milling of AZ91D alloy [18]. The protective coatings used in the research work include: TiN coatings [17], TiAlN [19], TiB_2_ [12] and TiAlCN.

Based on the professional literature, it can be noticed that in terms of modelling, scientists most often conduct research in the field of optimisation and prediction of surface roughness using, among others, the Multiple Regression Technique [20,21,22], Taguchi Analysis [23,24,25] or Artificial Neural Networks [26,27,28]. Based on the works related to the use of ANNs given in Table 1, it can be concluded that the research works conducted by scientists relate to various processes of turning, milling and abrasive water jet machining (AWJM). Various materials were analysed in these works, i.e., magnesium alloys [29,30,31,32], aluminium alloys [28,33,34,35,36,37], titanium alloys [26,38,39], nickel alloys [20], cobalt alloys [40] and steel [41,42].

Based on the comparison of modelling methods using ANNs for the surface roughness parameter, it is clearly seen that researchers in the field of turning and milling primarily deal with modelling only one surface roughness parameter, which is the Ra parameter. It seems that such a narrow scope of research is insufficient to carry out a detailed analysis of surface conditions, as mentioned above. Few researchers have attempted to model more than one parameter. One of such works is the study by Zerti et al. [28], who performed dry turning simulations and extended their analyses with the Rz and Rt parameters. Moreover, the authors of this study [30] performed surface roughness tests (Ra, Rz, RSm) after milling AZ91D magnesium alloy using PCD inserts.

Based on the analysis of the literature on the machinability of magnesium alloys and the possibility of modelling roughness parameters, it can be concluded that:

- thanks to excellent properties and good machinability, the use of magnesium alloys is constantly expanded, especially where high mechanical strength and low specific weight are needed,
- it is justified to extend the research to expand the range of the technological parameters; however, one should take into account the tendency of magnesium alloys to spontaneous combust with a sudden increase in temperature,
- surface roughness is estimated using a small number of parameters, usually Ra and Rz—there is no wider analysis of a larger group of surface roughness parameters, including a more detailed analysis of the features and operational properties of the machined surface,
- the research works conducted so far have referred only to roughing.

Therefore, the aim of this study is to determine the 2D surface roughness for AZ91D and AZ31 magnesium alloys after finish milling. The influence of the change of machining parameters and the change of the helix angle was analysed. The fact that there is no publication on the finish machining of magnesium alloys, which would constitute a significant supplement to the previous research works usually focused on the conditions of high-speed, effective roughing, can be considered a novelty.

## 2. Materials and Methods

### 2.1. Testing Methodology

The object of the research was two magnesium alloys: AZ91D and AZ31. Milling was carried out in three sets of cutting parameters for both the lateral face and the end face of the tested samples, in the following ranges: cutting speed v_c_ = 400–1200 m/min, feed per tooth f_z_ = 0.05–0.3 mm/tooth, axial depth of cut a_p_ = 0.1–0.5 mm and radial depth of cut a_e_ = 0.5–3.5 mm. The surface roughness parameters analysed were: Ra, Rz, RSm, Rsk and Rku. Two carbide cutters were applied in the research:

- triple-edge milling cutter: Ø16 mm, tool cutting edge helix angle λ_s_ = 20°, tool rake angle γ = 12°, tool clearance angle α = 8°,
- triple-edge milling cutter: Ø16 mm, tool cutting edge helix angle λ_s_ = 50°, tool rake angle γ = 15°, tool clearance angle α = 8°.

**Figure 1:**
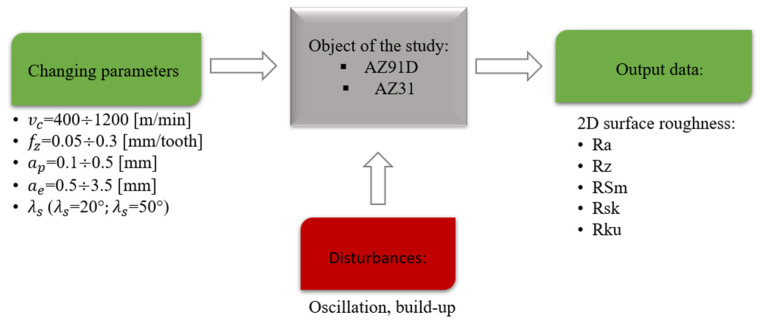
Research schematic diagram of the test set-up.

The choice of tools was determined by verifying the influence of the tool cutting edge helix angle (inclination angle) on the surface roughness. The research schematic diagram of the test set-up is given in Figure 1.

**Figure 2:**
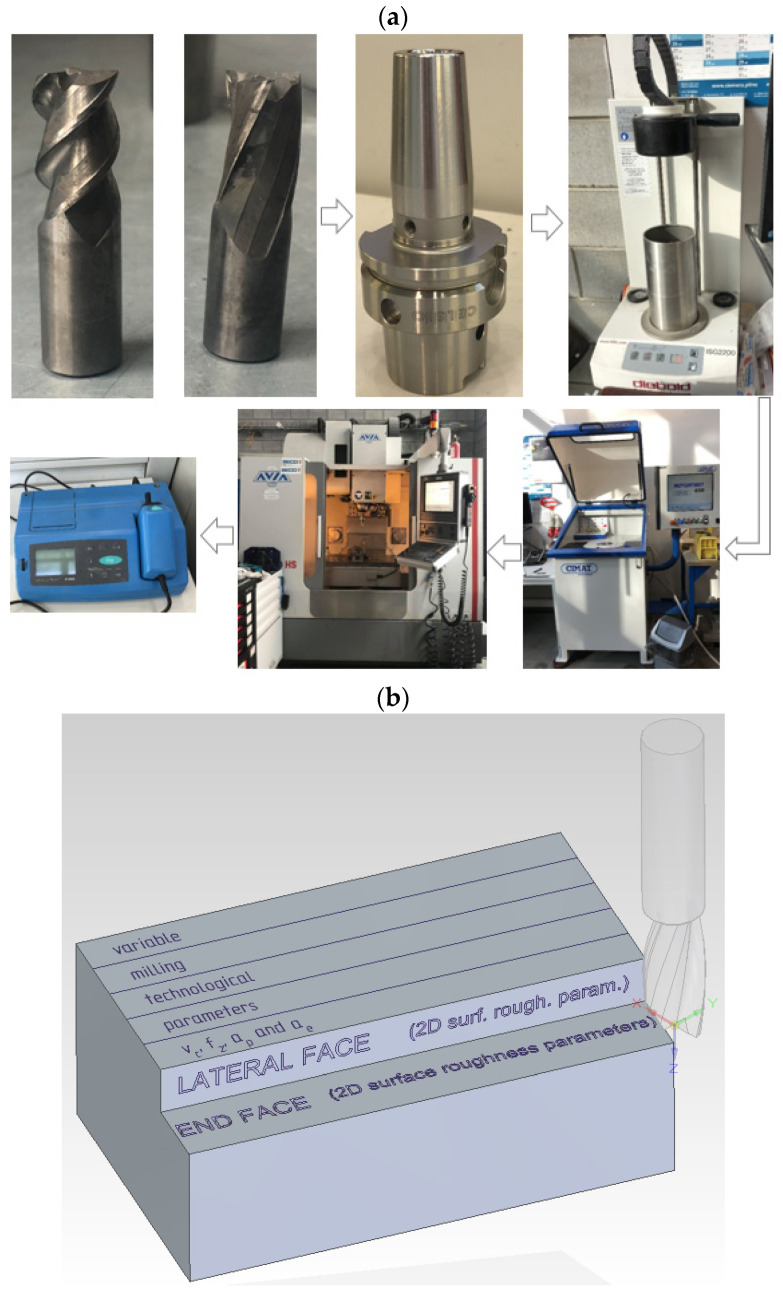
Schematic diagram of the test stand: (**a**) object of the study, and (**b**) visualisation with the roughness measurement model on the end face and the lateral face of the magnesium alloys specimens.

The cutters were mounted into a CELSIO HSK-A63 16 × 95 tool holder from SCHUNK (Lauffen am Neckar, Germany) using an ISG 2200 shrink fit tool holder machine from H. Diebold GmbH & CO (Jungingen, Germany). The tool with the holder was balanced dynamically by a CIMAT RT 610 balancing machine (Bydgoszcz, Poland), according to ISO 21940–11:2016 balancing class G 2.5. The measured value of the residual unbalance was 0.25 gmm. Milling was performed on an AVIA VMC 800 HS-type vertical machining centre, equipped with the Heidenhain iTNC 530 control system, with a maximum spindle speed of 24,000 rpm. The schematic diagram of the test stand is given in Figure 2. Figure 2a presents the object of the study. Figure 2b shows the visualisation with the roughness measurement model. The quality of the end face and the lateral face of the AZ31 and AZ91D magnesium alloys specimens was described using 2D roughness parameters.

The surface roughness measurements were carried out using a HOMMEL TESTER T1000 contact profilometer from ITA K Pollak M Wieczorowski Sp. J. (Poznań, Poland). The measurement parameters were: traverse length lt = 4.8 mm, sampling length lr = 0.8 mm, scanning rate v_t_ = 0.5 mm/s and measuring range/resolution M = ±320 µm/0.04 µm. Each measurement was repeated five times on each surface towards the assessment of the mean values and standard deviation.

### 2.2. Statistical Analysis Methodology

**Figure 3:**
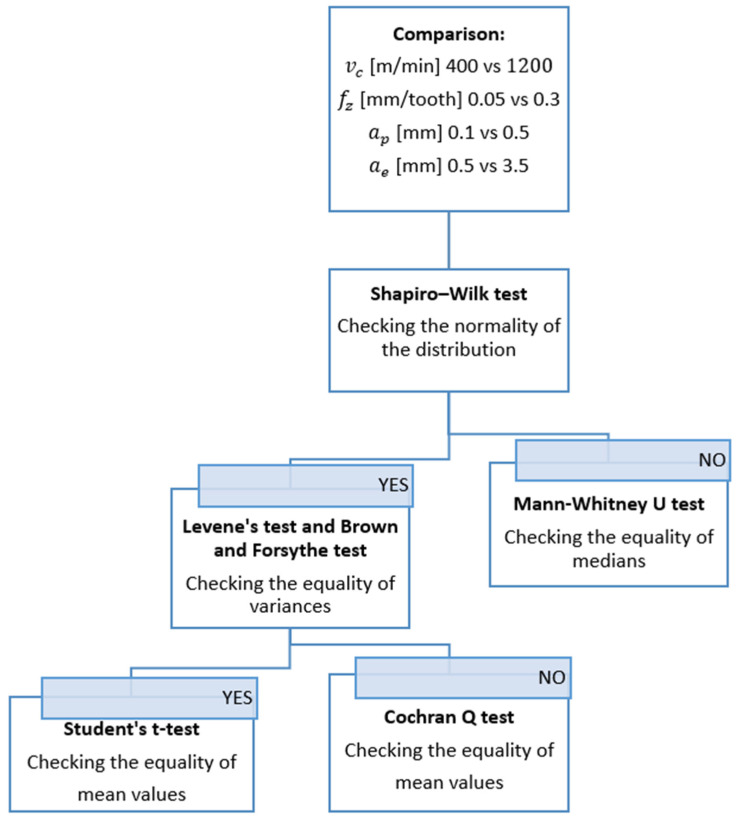
Statistical test selection scheme [43].

The data obtained from the roughness measurements were subjected to statistical verification. The output data obtained in the study were treated as independent, quantitative variables. The significance level of α = 0.05 was assumed. The normality of distribution was examined, and the hypotheses of the equality of mean values and variance were verified. The fulfilment of specified assumptions regarding the data was the basis for the decision to select the test [43]. In selecting the appropriate statistical test to assess the truth of the hypothesis, a simplified scheme was used, which is given in Figure 3. All statistical tests were performed using the Statistica 13.3 software (TIBCO Software Inc., Palo Alto, CA, USA).

Firstly, the Shapiro–Wilk test was performed to examine the normality of the distribution [43]. The null hypothesis and the alternative hypothesis were as follows:

- *H*_0_: The distributions are not different from the normal distribution in a statistically significant way,
- *H*_1_: The distributions are different from the normal distribution in a statistically significant way.

When the normality condition was not met, the Mann–Whitney U test was employed for further verification. The Mann–Whitney U test is used to check the equality of the medians [43]. The null hypothesis and the alternative hypothesis were as follows:

(1) $$H_{0}:Me_{1}=Me_{2}$$

(2) $$H_{1}:Me_{1}\neq Me_{2}$$

If the case with normal distribution was confirmed, the hypothesis of equality of variances was checked by Levene’s test and the Brown–Forsythe test [36]. The null hypothesis and the alternative hypothesis were as follows:

(3) $$H_{0}:\;\sigma_{1}{}^{2}=\sigma_{2}{}^{2}$$

(4) $$H_{1}:\;\sigma_{1}{}^{2}\neq \sigma_{2}{}^{2}$$

In two cases, the hypothesis about the equality of mean values was verified. In the absence of grounds for rejecting the null hypothesis, Student’s *t*-test was used to verify the hypothesis of the equality of mean values. If the condition of homogeneity of variance had not been met, the Cochran’s Q test would have been used to verify the hypothesis of the equality of mean values [43]. The null hypothesis and the alternative hypothesis were as follows:

(5) $$H_{0}:\mu_{1}{}^{2}=\mu_{2}{}^{2}$$

(6) $$H_{1}:\;\mu_{1}{}^{2}\neq \mu_{2}{}^{2}$$

### 2.3. Artificial Neural Network Methodology

**Figure 4:**
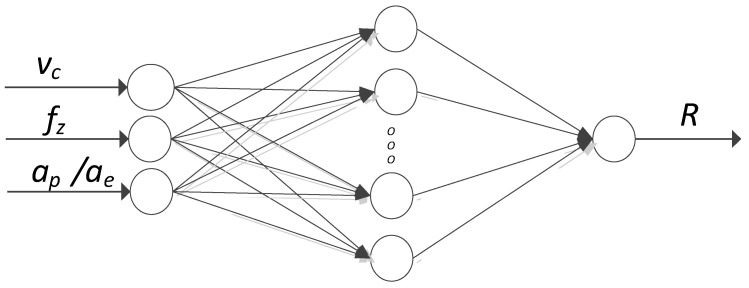
Artificial neural networks scheme.

For the AZ91D magnesium alloy on the lateral and the end faces (Ra, Rz parameters), simulations were carried out using artificial neural networks. The scheme of the artificial neural networks is shown in Figure 4, where R in the output denotes the corresponding surface roughness parameter: a_p_ axial or a_e_ radial depth of cut [30].

The input layer consists of input neurons that are equal to the number of input data: cutting speed v_c_, feed per tooth f_z_ and axial depth of cut a_p_ for data obtained from the surface roughness measurements on the end face of the tested sample. For the lateral surface, the variable factors were cutting speed v_c_, feed per tooth f_z_ and radial depth of cut a_e_.

Two types of ANN were used: MLP (Multi-layered perceptron) and RBF (Radial Basis Function). The possible activation functions used in the MLP model are as follows: linear, logistic, tanh, exponential, sine. The MLP model was trained using the BFGS learning algorithm. The RBF network model was trained with the RBFT algorithm and included the following activation functions: hidden neurons—Gaussian distribution, output neurons—linear function.

The number of random samples was as follows: 75% training, 25% validation and testing was overlooked due to the small amount of all data sets [44].

Moreover, the following parameters were experimentally determined: the number of hidden neurons (2–10) and the number of epochs (150–300). The assessment of individual networks was executed on the basis of a comparison of the training quality, the quality of validation and the learning error determined by the least-squares method [45].

### 2.4. Objective of Research and Novelties

The presented work presents an analysis of the surface roughness after finish milling. The analysis of the literature shows that the vast majority of scientific publications concern the rough milling of magnesium alloys. Thus, the proposed research topic is innovative and new in the sense of originality of the scientific goal. One machinability factor is analysed in this work, namely the roughness of the 2D surface. This indicator is particularly important in the production processes of workpieces, both for scientific and cognitive reasons, as well as the possibility of implementing the presented research results (ranges of machining parameters) in industrial plants dealing with the subject of magnesium alloys machining. Moreover, after the analysis of the literature, it can be concluded that many researchers were mainly concerned with the analysis of the basic parameters of surface roughness, both 2D and 3D, ignoring the aspect of maintenance features of the treated surface. This state of affairs is definitely insufficient, hence the proposal to analyse additional roughness parameters, such as RSm, Rsk and Rku. It is a challenge of some kind to be able to predict specific performance properties of the parts produced on the basis of the roughness parameters correlated with the functional properties of the surface. Apart from the main goal (defining the influence of cutting parameters on the change of roughness parameters), the modelling of selected surface roughness parameters (Ra, Rz) can be presented as an additional goal in order to forecast these parameters.

Modelling the roughness parameters after milling can be the basis for creating tools helpful in the work of the technologist for determining the conditions of the machining process in order to obtain the assumed surface roughness. Thus far, no such works have been carried out after the finish milling of magnesium alloys. In addition, the use of, inter alia, artificial neural networks can contribute to the reduction of the number of research trials necessary for the selection and optimisation of the parameters of the technological process. Additionally, the conducted analysis may be interesting due to the universality and availability of both the tools (end mills) and the presented magnesium alloys.

## 3. Results and Discussion

### 3.1. Surface Roughness

**Figure 5:**
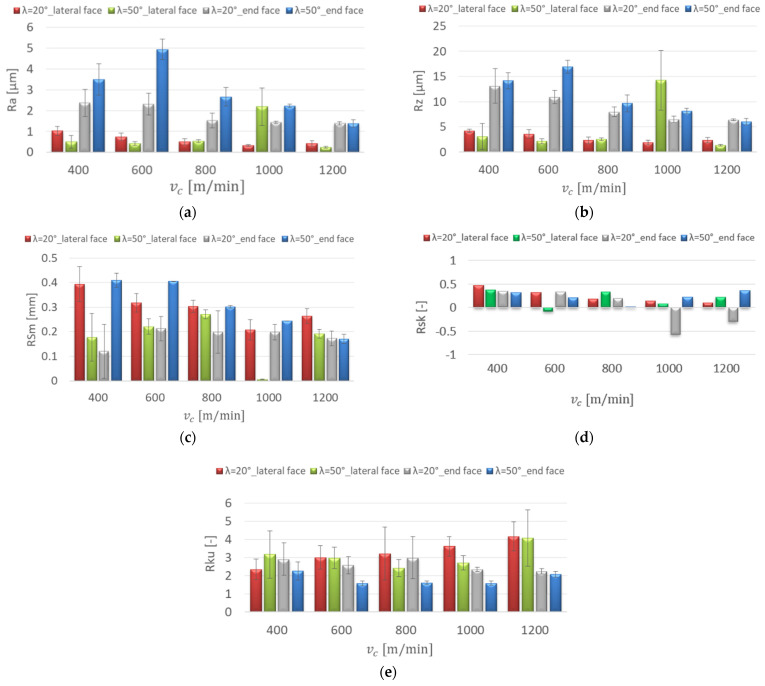
Effect of cutting speed change on the surface roughness: (**a**) Ra, (**b**) Rz, (**c**) RSm, (**d**) Rsk, (**e**) Rku; f_z_ = 0.15 mm/tooth, lateral face: a_e_ = 2 mm, a_p_ = 8 mm, end face: a_e_ = 14 mm, a_p_ = 0.3 mm.

The surface roughness of AZ91D and AZ31 magnesium alloys after milling was analysed depending on the changes in technological parameter values and tools with different tool cutting edge helix angles. Figure 5 shows the influence of the cutting speed on the roughness parameters for the AZ91D magnesium alloy.

The diagrams presented in Figure 5a,b prove that regardless of the tool used, an increase in cutting speed v_c_ resulted in decreasing Ra and Rz values, especially in the cutting speed range 600–1200 m/min. The lowest value for the parameters Ra = 0.235 μm and Rz = 1.37 μm was obtained for the λ_s_ = 50° tool, with v_c_ = 1200 m/min on the lateral face of the workpiece. The highest value of the parameters Ra = 4.941 μm and Rz = 16.95 μm was observed at v_c_ = 600 m/min for the λ_s_ = 50° tool on the end face of the workpiece.

For the λ_s_ = 50° tool, the value of the RSm parameter on the lateral face at v_c_ = 1000 m/min was 0.006 mm, which was much lower than the other values on the end face and for the λ_s_ = 20° tool, which ranged between 0.12 and 0.409 mm (Figure 5c). The value of the Rsk (skewness) below 0 is characterised by a higher frequency of deep valleys on the surface, which was obtained at higher cutting speeds: v_c_ = 1000, 1200 m/min for the λ_s_ = 20° tool on the end face of the specimens and for the λ_s_ = 50° tool on the lateral face at v_c_ = 600 m/min (Figure 5d). The distribution of profile roughness was close enough to the Gaussian distribution at the cutting speed v_c_ = 600 m/min on the lateral face of the workpiece for the λ_s_ = 50° tool (Rku = 2.976) and for the λ_s_ = 20° tool (Rku = 3.007) and on the end face at v_c_ = 800 m/min, the value of Rku was 2.991 (λ_s_ = 20°) (Figure 5e).

**Figure 6:**
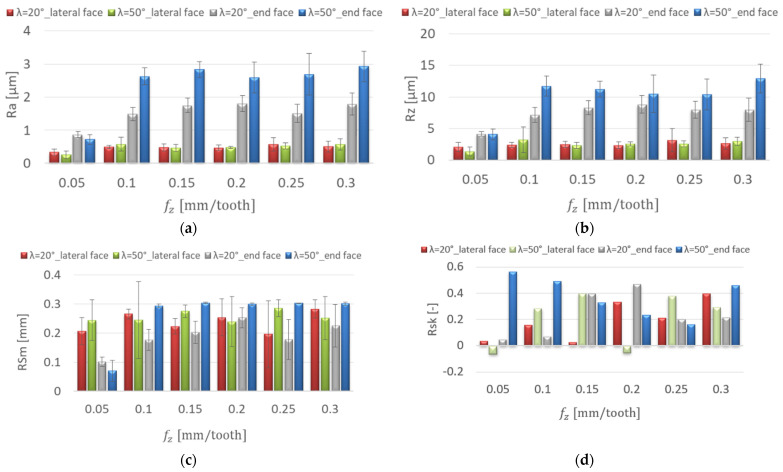

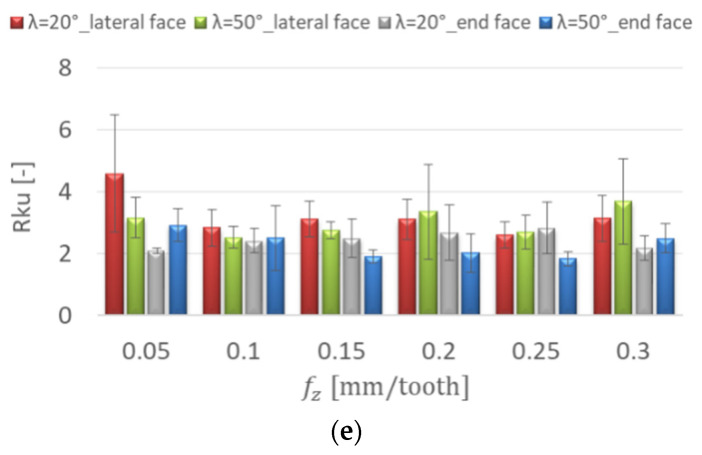
Effect of feed per tooth f_z_ change on the surface roughness: (**a**) Ra, (**b**) Rz, (**c**) RSm, (**d**) Rsk, (**e**) Rku; v_c_ = 800 m/min, lateral face: a_e_ = 2 mm, a_p_ = 8 mm, end face: a_e_ = 14 mm, a_p_ = 0.3 mm.

Figure 6 shows the effect of the feed per tooth change on the surface roughness for the AZ91D magnesium alloy. In the range of feed per tooth f_z_ = 0.05–0.3 mm/tooth, lower Ra and Rz values were obtained on the lateral face, and their increase was not as pronounced as on the end face of the workpiece. The highest values of Ra = 2.924 μm and Rz = 12.9 μm were observed on the end face of the specimens for the λ_s_ = 50° tool at f_z_ = 0.3 mm/tooth. On the lateral face, the cutting-edge helix angle of the tool did not have a significant influence on the Ra and the Rz values, which were similar and in the range of Ra = 0.259–0.578 μm and Rz = 1.36–3.23 μm. However, the lowest values of the Ra and Rz parameters were obtained at f_z_ = 0.05 mm/tooth for the λ_s_ = 50° tool (Figure 6a,b). The values of the RSm parameter on the lateral face were in the range of 0.196–0.285 mm and showed insignificant change with increasing feed per tooth. On the end face of the workpiece (λ_s_ = 50° tool), a radical increase in RSm (by 0.223 mm) was observed with the change from f_z_ = 0.05 to 0.1 mm/tooth (Figure 6c). On the lateral face, Rsk (skewness) had positive and negative values. The symmetric profile distribution is expressed as Rsk = 0. Positive values of the Rsk coefficient were obtained on the end faces. These surfaces are marked by good resistance to adhesion (Figure 6d). The values of the Rku coefficient showed insignificant change with the increase of the feed per tooth. Kurtosis values below 3 were obtained on the end face of the specimens. These surfaces were characterised by greatly rounded peaks and valleys, which resulted in an increase in the friction coefficient (Figure 6e).

**Figure 7:**
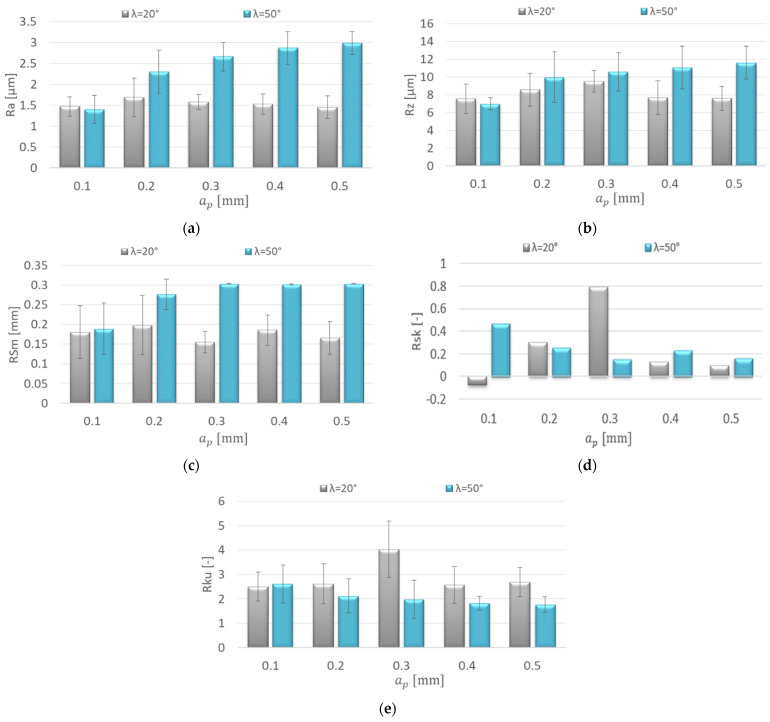
Effect of axial depth of cut a_p_ change on the surface roughness: (**a**) Ra, (**b**) Rz, (**c**) RSm, (**d**) Rsk, (**e**) Rku; v_c_ = 800 m/min, f_z_ = 0.15 mm/tooth, a_e_ = 14 mm.

Figure 7 shows the effect of increasing the axial depth of cut a_p_ on the roughness parameters for the AZ91D magnesium alloy. Similar values of the Ra parameter were obtained in the entire range of the axial depth of cut for the λ_s_ = 20° tool, which ranged from 1.453 to 1.687 μm. The axial depth of cut change also did not have a significant impact on the Rz parameter value (7.55–9.51 μm). With the range a_p_ = 0.2–0.5 mm, for the λ_s_ = 50° tool, higher values of the mentioned parameters were obtained, which were in the following ranges: Ra = 1.402–2.987 μm and Rz = 6.96–11.6 μm (Figure 7a,b).

The increase in the axial depth of cut did not show a dramatic change in the values of the RSm parameter. For the λ_s_ = 20° tool, the RSm values ranged between 0.155 and 0.198 mm and were lower compared to the λ_s_ = 50° tool (0.189–0.303 mm). The differences in the values of the RSm parameter depending on the tool used were especially prevalent at a_p_ = 0.2–05 mm (Figure 7c). In the entire range of the axial depth of cut, the Rsk (skewness) values are small, close to the symmetric distribution of the profile. Rsk was only higher compared to the others for the λ_s_ = 20° tool with a_p_ = 0.3 mm; the discussed values do not exceed 0.8 (Figure 7d). Kurtosis values below 3 (Rku < 3) were obtained for almost the entire range of the axial depth of cut. The Rku value was only greater than 3 for the λ_s_ = 20° tool at a_p_ = 0.3 mm (Figure 7e).

**Figure 8:**
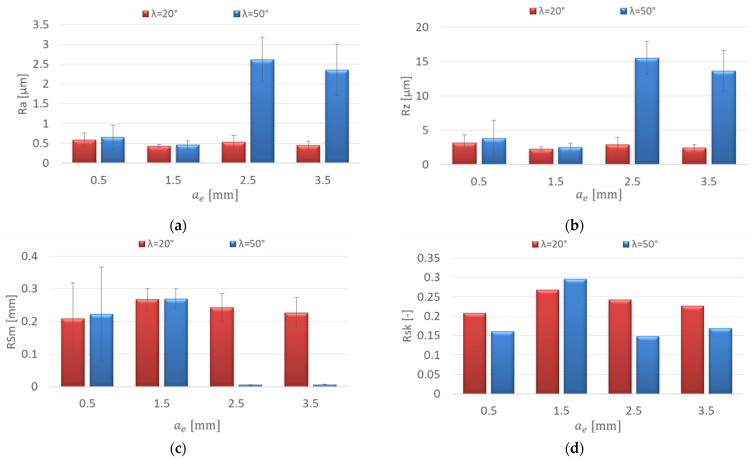

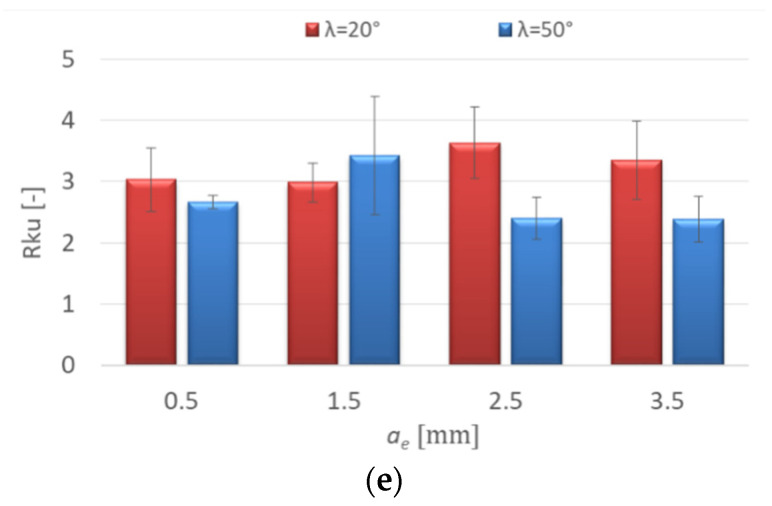
Effect of radial depth of cut a_e_ change on the surface roughness: (**a**) Ra, (**b**) Rz, (**c**) RSm, (**d**) Rsk, (**e**) Rku; v_c_ = 800 m/min, f_z_ = 0.15 mm/tooth, a_p_ = 8 mm.

Figure 8 shows the influence of the increasing radial depth of cut a_e_ on the roughness parameters for the AZ91D magnesium alloy. The research showed an insignificant effect of the increase in the radial depth of cut on the roughness parameters: Ra = 0.431–0.589 μm and Rz = 2.28–3.14 μm for the λ_s_ = 20° tool. On the other hand, for the λ = 50° tool, a dramatic increase in the Ra (by 2.151 μm) and Rz parameters (by 12.99 μm) was observed with the change of the radial depth of cut from a_e_ = 1.5 to 2.5 mm. When increasing to a_e_ = 3.5 mm, the parameter values did not change significantly (Figure 8a,b). For the λ_s_ = 20° tool, it was shown that the change in the radial depth of cut had a negligible effect on the RSm parameter values, which were in the range 0.208–0.268 mm. The values of the RSm parameter after milling with the λ_s_ = 50° tool with a_e_ = 2.5–3.5 mm were several times lower than those obtained with smaller values of a_e_. (Figure 8c). Positive values of Rsk (skewness) were observed in the entire range of the radial depth of cut (Figure 8d). The kurtosis (Rku) values oscillated around 3 for both tools in the entire range of the radial depth of cut, which means that the distribution of the roughness profile corresponded to the Gaussian distribution (Rku = 2.381–3.633) (Figure 8e).

**Figure 9:**
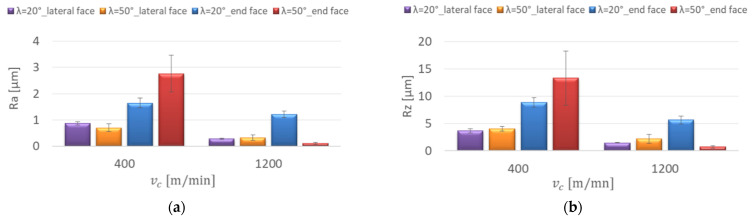

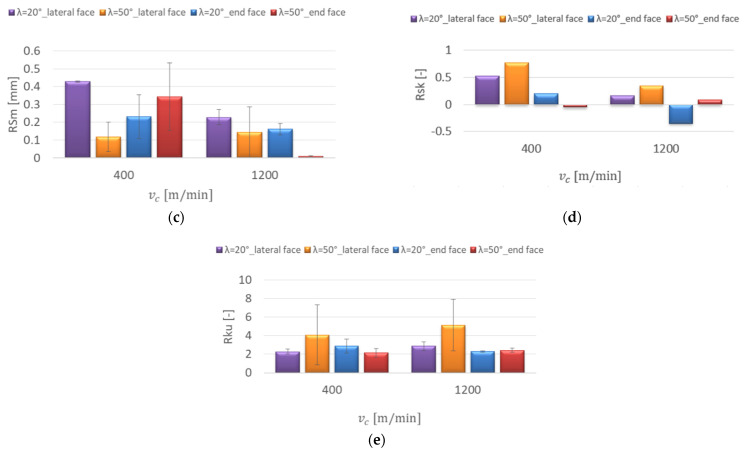
Effect of cutting speed change on the surface roughness: (**a**) Ra, (**b**) Rz, (**c**) RSm, (**d**) Rsk, (**e**) Rku; f_z_ = 0.15 mm/tooth, lateral face: a_e_ = 2 mm, a_p_ = 8 mm, end face: a_e_ = 14 mm, a_p_ = 0.3 mm.

Figure 9 shows the influence of the cutting speed on the roughness parameters for the AZ31 magnesium alloy. For this alloy, the surface roughness was analysed with the extreme values of the cutting parameters.

When analysing the effect of cutting speed v_c_, lower values were clearly observed at v_c_ = 1200 m/min on the lateral and end faces for both tools. The greatest influence of the cutting speed increase on the values of the Ra and Rz parameters was apparent for the λ_s_ = 50° tool. With the cutting speed v_c_ = 400 m/min, the highest values were obtained on the end face (Ra = 2.764 μm, Rz = 13.32 μm). With the increase of the cutting speed to v_c_ = 1200 m/min, the Ra parameter value decreased by 2.647 μm and the Rz parameter value decreased by 12.61 μm (Figure 9a,b). At the cutting speed v_c_ = 400 m/min, the RSm parameter values were in the range of 0.117–0.428 mm. With the cutting speed v_c_ = 1200 m/min for the λ_s_ = 50° tool on the end face, a radical decrease in the value of the RSm parameter was achieved to 0.008 mm, which was several times lower compared to the other values. In addition, a greater dispersion of the results in the series of measurements after milling with the λ_s_ = 50° tool was noticed, especially for the surface roughness parameters on the lateral face of the workpiece (Figure 9c).

The Rsk (skewness) values on the end face were small, close to the symmetric distribution of the profile roughness. On the lateral face, lower Rsk values were obtained at a higher cutting speed (v_c_ = 1200 m/min). The values for both the lateral and the end faces did not exceed 0.8 (Figure 9d). The values of the Rku (kurtosis) after milling with the λ_s_ = 20° tool ranged from 2.272 to 2.879. On the other hand, for the λ_s_ = 50° tool, lower values of the coefficient (Rku < 3) were noticed on the end face. Rku (kurtosis) values greater than 3 were obtained on the lateral face at v_c_ = 400 m/min (4.068) and at v_c_ = 1200 m/min (5.133), which is characteristic for surfaces with sharp peaks and valleys (Figure 9e).

**Figure 10:**
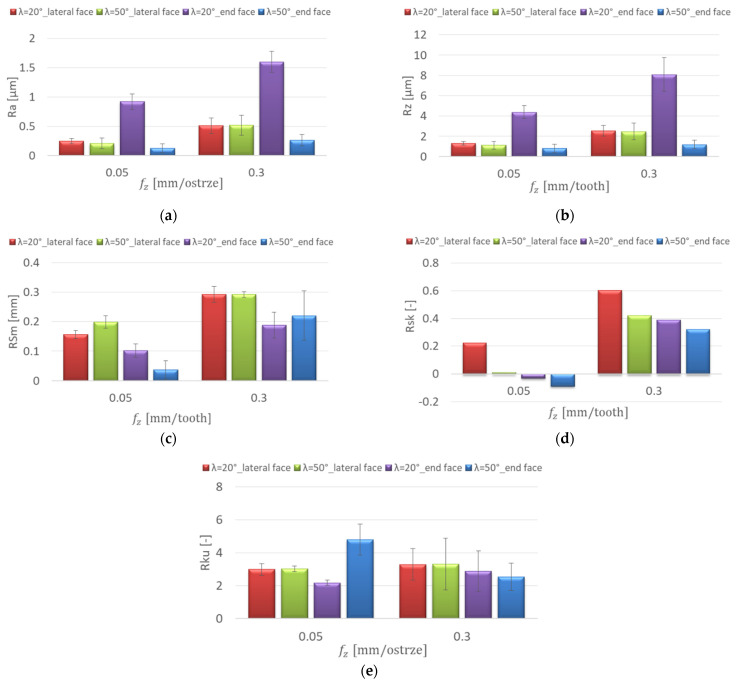
Effect of feed per tooth f_z_ change on the surface roughness: (**a**) Ra, (**b**) Rz, (**c**) RSm, (**d**) Rsk, (**e**) Rku; v_c_ = 800 m/min, lateral face: a_e_ = 2 mm, a_p_ = 8 mm, end face: a_e_ = 14 mm, a_p_ = 0.3 mm.

Figure 10 shows the effect of the feed per tooth change on the surface roughness for the AZ31 magnesium alloy. In the research, increasing the feed per tooth f_z_ increased the values of the Ra and the Rz parameters; it was especially visible on the end face for the λ_s_ = 20° tool, where the value of the Ra parameter increased from 0.919 to 1.6 μm and the value of the Rz parameter from 4.37 to 8.09 μm. The lowest values of the Ra and the Rz parameters were observed on the end face for the λ_s_ = 50° tool at f_z_ = 0.05 mm/tooth (Figure 10a,b). The change of the feed per tooth from 0.05 to 0.3 contributed to an increase in the value of the RSm parameter. The greatest difference was noted on the end face of the workpiece for the λ_s_ = 50° tool, where the RSm value increased from 0.037 to 0.221 mm (Figure 10c). The values of the Rsk coefficient with the feed per tooth f_z_ = 0.05 mm/tooth oscillated around 0. With the feed per tooth f_z_ = 0.3 mm/tooth, positive values of the Rsk coefficient were obtained for the two tools (Figure 10d). The values of the Rku coefficient oscillated around 3, which shows that the profile distribution corresponded to the Gaussian distribution. Only the value of the Rku coefficient on the end face of the workpiece after milling with the λ_s_ = 50° tool at a feed per tooth f_z_ = 0.05 mm/tooth was 4.796 (Figure 10e).

**Figure 11:**
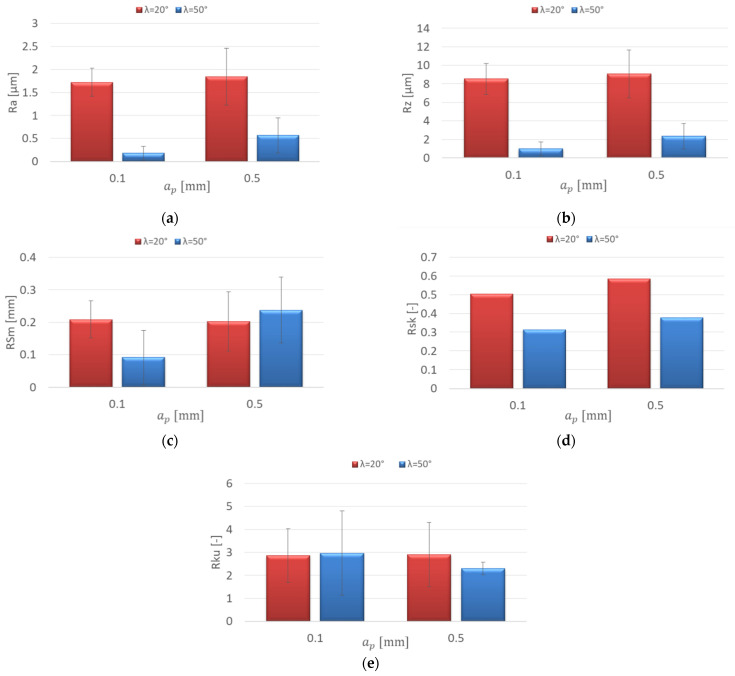
Effect of axial depth of cut a_p_ change on the surface roughness: (**a**) Ra, (**b**) Rz, (**c**) RSm, (**d**) Rsk, (**e**) Rku; v_c_ = 800 m/min, f_z_ = 0.15 mm/tooth, a_e_ = 14 mm.

Figure 11 shows the effect of increasing the axial depth of cut a_p_ on the roughness parameters for the AZ31 magnesium alloy. For the λ_s_ = 20° tool, the influence of the increase in the axial depth of cut a_p_ on the values of the Ra and Rz parameters was negligible; the values were in the following ranges: Ra = 1.721–1.844 μm and Rz = 8.53–9.07 μm. In the case of the λ_s_ = 50° tool, higher values of the discussed parameters were obtained with a_p_ = 0.5 mm. The value of the Ra parameter increased by 0.386 μm, and the Rz parameter increased by 1.35 μm (Figure 11a,b). It was also shown that the change in the axial depth of cut a_p_ for both tools had an insignificant effect on the values of the RSm parameter. For the λ_s_ = 20° tool, the RSm parameter value decreased by 0.006 mm, and for the λ_s_ = 50° tool, it increased by 0.146 mm (Figure 11c). Regardless of the increase in the axial depth of cut a_p_ and tool cutting edge helix angle (λ_s_ = 20°, λ_s_ = 50°), positive values of the Rsk coefficient were obtained (Rsk = 0.314–0.585) (Figure 11d). The values of the Rku coefficient were in the range: 2.296–2.969 (Figure 11e).

**Figure 12:**
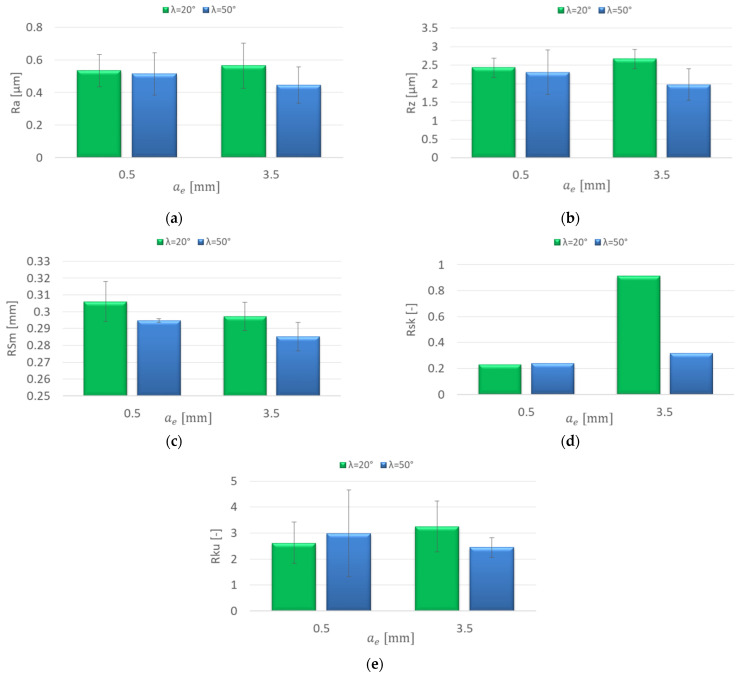
Effect of radial depth of cut a_e_ change on the surface roughness: (**a**) Ra, (**b**) Rz, (**c**) RSm, (**d**) Rsk, (**e**) Rku; v_c_ = 800 m/min, f_z_ = 0.15 mm/tooth, a_p_ = 8 mm.

Figure 12 shows the influence of increasing the radial depth of cut a_e_ on the roughness parameters for the AZ31 magnesium alloy. The effect of increasing the radial depth of cut a_e_ was negligible for the Ra and the Rz parameters; the values were similar regardless of the tool helix angle. The Ra parameter values were in the range from 0.455 to 0.565 μm. The Rz parameter values were in the range of 1.98–2.66 μm (Figure 12a,b). In the case of the RSm parameter, it was also shown that the change in radial depth of cut a_e_ had an insignificant effect. The parameter values ranged from 0.285 to 0.306 mm (Figure 12c). With a variable radial depth of cut, the Rsk (skewness) values were positive. The change of the radial depth of cut from a_e_ = 0.5 to 3.5 mm resulted in an increase in the Rsk coefficient value by 0.684 for the λ_s_ = 20° tool. For the λ_s_ = 50° tool, despite the increase in the radial depth of cut, the values of the Rsk coefficient were small, close to the symmetrical distribution of the profile and ranged between 0.228 and 0.315 (Figure 12d). The Rku (kurtosis) values were in the range of 2.446–3.258, which means that the roughness profile distributions were similar to the Gaussian distribution (Figure 12e).

### 3.2. Statistical Analysis

**Figure 13:**
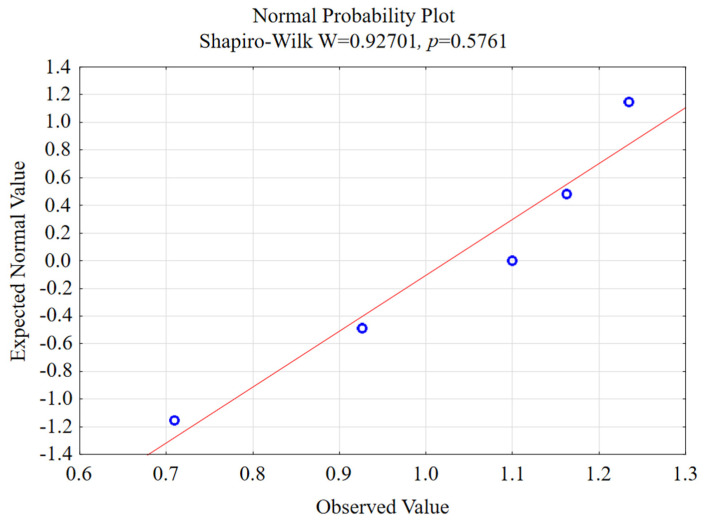
Normal probability plot for the λ_s_ = 20° tool with the analysis of the Ra parameter on the lateral face of the AZ91D magnesium alloy v_c_ = 400 m/min, f_z_ = 0.15 mm/tooth, a_e_ = 2 mm, a_p_ = 8 mm.

One of the conditions for using the test to check the equality of two means is the normality of the distribution, so firstly, the Shapiro–Wilk test was performed. Figure 13 shows the normal probability plot for selected machining conditions. Furthermore, Table 1, Table 2, Table 3 and Table 4 present a summary of the performed statistical test for the data obtained as a result of the studies carried out.

In the case of the *p*-value below 0.05, the distributions are different from the normal distribution in a statistically significant way. The *p*-value is the lowest significance level that leads to the rejection of the null hypothesis [46]. A *p*-value below 0.05 was obtained for the following parameters (Table 2):

- RSm for the variable v_c_ in the case of the lateral face,
- Rku for the variable f_z_ for the end face.

In Table 3, on the lateral face of the AZ91D magnesium alloy (λ_s_ = 50° tool), the roughness parameters that obtained a *p*-value below 0.05 were as follows: Rz, RSm and Rku with the variable v_c_, and Ra and Rz with the variable f_z_. Further, on the end face: Ra, RSm and Rsk with the variable f_z_. In Table 4, for the λ_s_ = 20° tool on the end face of the AZ31 magnesium alloy, a *p*-value below 0.05 was only obtained for the Ra parameter for the variable f_z_.

A *p*-value below 0.05 was obtained for the following parameters (Table 5):

- Lateral face of the workpiece: Rku for the variable v_c_, and Rku for the variable f_z_.
- End face of the workpiece: RSm and Rsk for the variable v_c_.

Therefore, the non-parametric Mann–Whitney U test was used for further analysis. Table 6 shows the test results for the data obtained in the study. For the roughness parameters, which obtained *p*-values greater than 0.05 (in the Shapiro–Wilk test), the hypothesis of the equality of variances was tested for further statistical verification. On the AZ91D lateral face (λ_s_ = 20° tool), in the case of the RSm roughness parameter with the variable v_c_, the observed difference was statistically significant. This means that the above-mentioned parameter was influenced by the cutting speed (lower values of these parameters were obtained at v_c_ = 1200 m/min). For the λ_s_ = 50° tool with the variable v_c_, the differences were statistically insignificant, although the registered *p*-value (*p* = 0.095238) for the Rz parameter was on the edge of statistical significance. However, with the variable f_z_ for the parameters Ra and Rz, it was proven that the differences were statistically significant, which means that the increase in feed per tooth adversely affects the mentioned parameters. On the end face of the alloy AZ91D (λ_s_ = 50° tool), the differences in the roughness parameters (Ra, RSm) for variable f_z_ were statistically significant.

For the λ_s_ = 20° tool on the end face of the AZ31 magnesium alloy, it was shown that an increase in feed per tooth from 0.05 to 0.3 mm tooth influenced the Ra parameter. For the λ_s_ = 50° tool on the lateral and end face of the AZ31 magnesium alloy, it was shown that the differences between the examined parameters were not statistically significant at the established significance level α = 0.05 (Table 6).

In order to further statistically verify the data for which the null hypothesis was not rejected (the distributions are not different from the normal distribution in a statistically significant way), it was necessary to check the equality of variances towards selecting the statistical test for checking the equality of the means [43].

Table 7, Table 8, Table 9, Table 10, Table 11, Table 12, Table 13 and Table 14 present the results of: Cochran’s Q test and Student’s *t*-test verifying the hypothesis of the equality of means.

Table 7 shows the results for the λ_s_ = 20° tool on the lateral face of the AZ91D alloy. It was shown that for the variable cutting speed, the differences were statistically significant for the Ra, Rz and Rku parameters. The increase in the cutting speed from v_c_ = 400 to 1200 m/min had a positive impact on the mean values of the mentioned parameters (the mean values of the Ra and Rz parameters were lower, at v_c_ = 1200 m/min). When changing the feed per tooth f_z_, only the differences for the RSm parameter were statistically significant. However, there was a *p*-value at the edge of statistical significance for the Ra parameter.

For the λ_s_ = 20° tool on the face of the AZ91D magnesium alloy, it was shown that the differences were only statistically insignificant for variable cutting speed for the RSm, and Rku parameters. The change in cutting speed from v_c_ = 400 to 1200 m/min had a positive effect on the mean values of the Ra, Rz and Rsk parameters. When changing the feed per tooth f_z_, it was shown that the mean values of the parameters Ra, Rz and RSm, were different in a statistically significant way. By increasing the feed per tooth from f_z_ = 0.05 to 0.3 mm/tooth, higher values of these parameters were obtained. The observed differences were only statistically insignificant for the Rsk coefficient (Table 8).

Table 9 shows the results of tests on the lateral face of the AZ91D alloy for the λ_s_ = 50° tool. The change in cutting speed v_c_ did not influence the mean values of the roughness parameters Ra and Rsk. However, there was a *p*-value on the verge of statistical significance for the Ra parameter. The observed differences in the mean values of the RSm, Rsk and Rku parameters when changing the feed per tooth f_z_ were not statistically significant. Nevertheless, it is worth highlighting the *p*-value (0.063011), which was on the edge of statistical significance for the Rsk coefficient.

Table 10 shows the results for the λ_s_ = 50° tool on the end face of the AZ91D alloy. The increase in the cutting speed from 400 to 1200 m/min had a positive effect on the values of the Ra, Rz and RSm parameters (the mean values of these parameters were lower, at v_c_ = 1200 m/min). When changing the feed per tooth f_z_, the mean value of the Rz parameter was different in a statistically significant way. By increasing the feed per tooth from f_z_ = 0.05 to 0.3 mm/tooth, a higher mean value of the parameter mentioned was obtained.

Table 11 shows the results of tests on the lateral face of the AZ31 alloy for the λ_s_ = 20° tool. It was shown that the differences were statistically significant for the roughness parameters Ra, Rz, RSm and Rku at the cutting speed change v_c_. The increase in the cutting speed from v_c_ = 400 to 1200 m/min had a positive influence on the mean values of the mentioned parameters. In the case of the Rsk coefficient, the differences were statistically insignificant at the established significance level. When changing the feed per tooth on the lateral face of the AZ31 alloy, it was shown that the observed differences were statistically significant. This means that the change of the feed per tooth f_z_ from 0.05 to 0.3 mm/tooth affected the mean values of the mentioned parameters (higher mean values of the Ra, Rz and RSm parameters were received at f_z_ = 0.3 mm/tooth).

Table 12 shows the test results for the AZ31 alloy end face (λ_s_ = 20° tool). The differences were statistically significant at the cutting speed change for the parameters Ra, Rz and Rsk. The increase in cutting speed from 400 to 1200 m/min had a positive effect on the mean values of these parameters (lower values were obtained at v_c_ = 1200 m/min). When changing feed per tooth f_z_, it was shown that the mean values of the Rz and RSm parameters were different in a statistically significant way. When increasing the feed per tooth from f_z_ = 0.05 to 0.3 mm/tooth, higher values of the given parameters were obtained.

On the lateral face of the AZ31 alloy (λ_s_ = 50° tool), the differences in the roughness parameters Ra and Rz with the variable v_c_ were statistically significant in favour of a higher cutting speed (lower values were obtained at v_c_ = 1200 m/min). At the feed per tooth change, it was shown that the mean values of the parameters Ra, Rz, RSm and Rsk were statistically different. By increasing the feed per tooth from 0.05 to 0.3 mm/tooth, higher values of these parameters were obtained (Table 13).

For the λ_s_ = 50° tool on the end face of the AZ31 magnesium alloy, it was shown that the differences in the mean values of the parameters Ra and Rz at variable cutting speeds v_c_ were statistically significant (lower roughness parameters were obtained at v_c_ = 1200 m/min). Only for the Rku coefficient, the mean values did not differ in a statistically significant way. At the feed per tooth change, the mean values of the RSm parameter and the Rku coefficient proved to be statistically significant. The other parameters with an increase in the feed per tooth f_z_ to 0.3 mm/tooth were not statistically different, although the *p*-value of 0.071161 received for Ra was at the edge of statistical significance (Table 15).

### 3.3. Artificial Neural Network

For the AZ91D magnesium alloy, simulations were carried out with the use of artificial neural networks in order to investigate the relationship between the technological parameters and the predicted surface roughness. The networks were created using 15 sets of technological parameters. For each input data, simulations were performed for 100 networks, while 10 networks were preserved for further analysis [45].

The network suitability was assessed by learning quality, validation quality, learning error and validation error. The simulations were carried out for the Ra and Rz parameters on the lateral and end faces of the workpiece. Table 14 shows the four different ANN models obtained. On the basis of the network quality indicators, it was shown that the best match for the Ra and Rz parameters on the lateral surface was obtained for the RBF 3-10-1 network (10 hidden neurons) while analysing the parameters on the end face of the AZ91D alloy showed that the MLP 3-2-1 network (2 hidden neurons) was optimal for the Rz parameter. However, for the Ra parameter, the RBF 3-9-1 network (9 hidden neurons) achieved a high quality of learning and validation in relation to its low error rate for learning and validation. For the MLP network, the best results were obtained for the exponential activation functions. The MLP 3-2-1 network for the Rz parameters on the end face of the AZ91D alloy was obtained after 9254 iterations.

**Figure 14:**
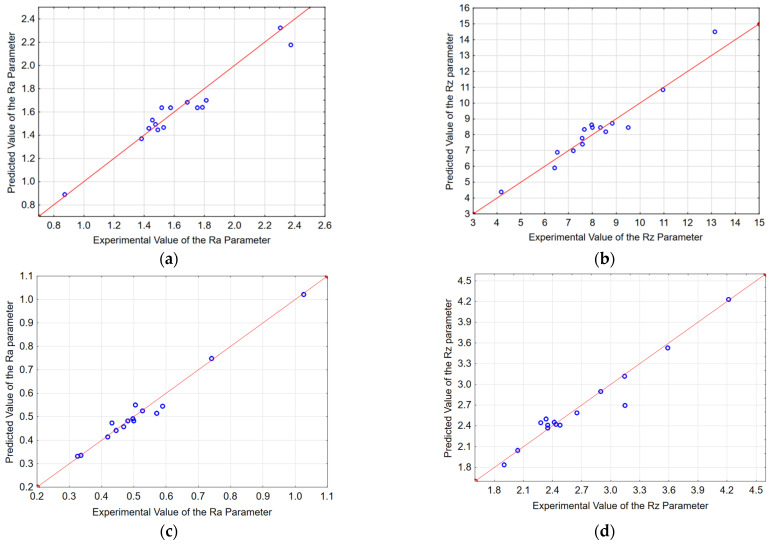
Comparison of predicted values with experimental values; end face (**a**) Ra, (**b**) Rz, lateral face: (**c**) Ra, (**d**) Rz.

The accuracy of the surface roughness prediction was assessed by comparing the predicted and experimental values. Figure 14 presents a visualisation of the accuracy of the prediction of the surface roughness parameters.

**Figure 15:**
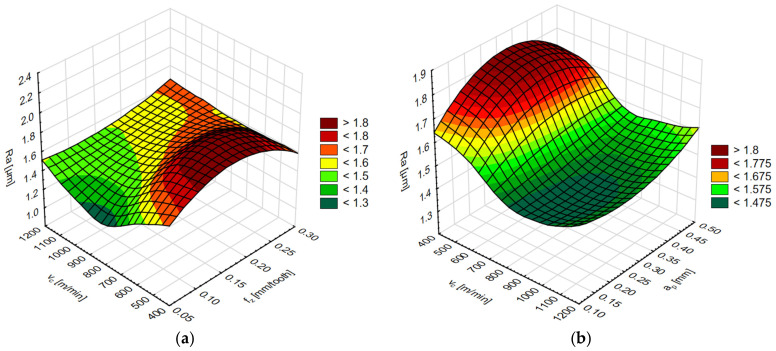
Simulation results of the Ra surface roughness parameter (**a**) for the variable cutting speed v_c_ and feed per tooth f_z_, and (**b**) for the variable cutting speed v_c_ and axial depth of cut a_p_.

**Figure 16:**
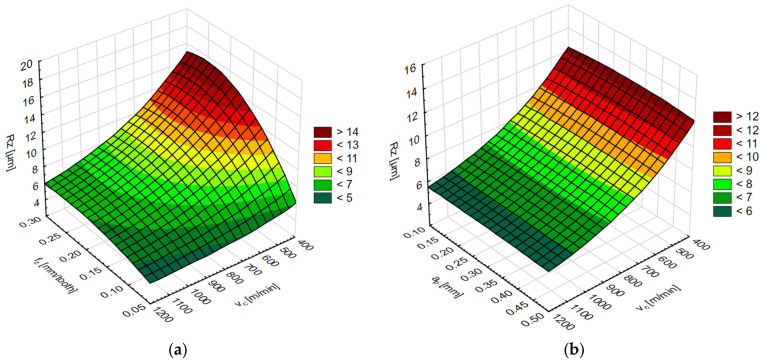
Simulation results of the Rz surface roughness parameter (**a**) for the variable cutting speed v_c_ and feed per tooth f_z_, and (**b**) for the variable cutting speed v_c_ and axial depth of cut a_p_.

**Figure 17:**
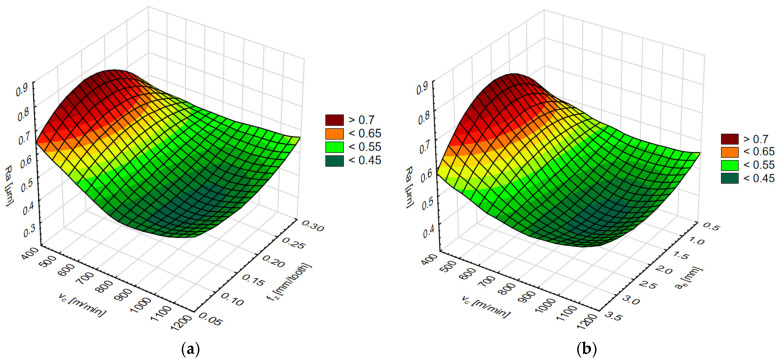
Simulation results of the Ra surface roughness parameter (**a**) for the variable cutting speed v_c_ and feed per tooth f_z_, and (**b**) for the variable cutting speed v_c_ and radial depth of cut a_e_.

**Figure 18:**
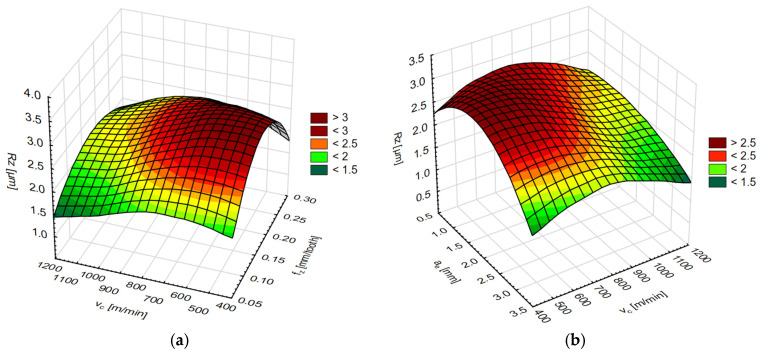
Simulation results of the Rz surface roughness parameter (**a**) for the variable cutting speed v_c_ and feed per tooth f_z_, and (**b**) for the variable cutting speed v_c_ and radial depth of cut a_e_.

The simulation results for each network are shown in two diagrams, depending on the cutting speed and feed per tooth or cutting speed and axial/radial depth of cut. The results of the surface roughness parameters on the end face of the AZ91D magnesium alloy are shown in Figure 15 and Figure 16 and on the lateral face in Figure 17 and Figure 18.

In order to determine whether each technological parameter affects the roughness parameter, a sensitivity analysis was performed (Table 16). None of the analysed technological parameters obtained a sensitivity analysis value below 1, which means that each of them had a significant impact. If you remove one of the v_c_, f_z_, a_p_ or a_e_ parameters, the network quality may be degraded. The cutting speed v_c_ was the parameter that had the strongest influence on the roughness parameters Ra and Rz on both the lateral and the end faces [47].

In summary, the relative error between the roughness parameter values from the ANN simulation and the experimental values did not exceed 15%. This means that the network quality was good enough to be used for simulation [37].

## 4. Conclusions

The conducted research and analyses allowed for the following conclusions:

1. The increase in the cutting speed has the greatest influence on the surface roughness. For both tools (λ_s_ = 20°, λ_s_ = 50°), a clear decrease in the roughness parameter values for both magnesium alloys AZ91D and AZ31 was observed.
2. Increasing feed per tooth reduces the surface quality; for the AZ91D magnesium alloy, a radical increase in the roughness parameters values was observed after the increase from f_z_= 0.05 to 0.1 mm/tooth. Within the range of f_z_ = 0.1–0.3 mm/tooth, no significant changes were observed.
3. The differences in roughness parameters depending on the tool used were clearly noticed after changing the radial and axial depth of cut.
4. Axial and radial depth of cut had a negligible influence on the values of the surface roughness parameters for the tool λ_s_ = 20° for both AZ31 and AZ91D alloys.
5. For the tool λ_s_ = 20°, differences were observed in the roughness parameters between the AZ91D and AZ31 alloys after changes in the cutting speed and the feed rate per tooth (lower roughness parameter values were obtained for AZ31).
6. Comparing the AZ91D and AZ31 alloy surface quality after milling using the tool λ_s_ = 50°, significant differences were found after changing the feed rate per tooth, the axial depth of cut (end face) and the radial depth of cut (lateral face). For the AZ31 magnesium alloy, lower parameter values were obtained, and less radical changes were observed from the increase in a given cutting parameter.
7. The statistical analysis of the roughness parameters Ra and Rz confirmed the inequality of the mean values and medians when changing the cutting speed v_c_. The increase in the cutting speed from 400 to 1200 m/min had a positive impact on the mentioned parameters (lower values of the surface roughness parameters were observed at the cutting speed v_c_ = 1200 m/min).
8. The statistical analysis shows that for all cases, the change of the feed per tooth f_z_ influenced the values of the Ra parameter. With the increase in the feed per tooth value from 0.05 to 0.3 mm/tooth, higher values of this parameter were obtained.
9. Comparing the tools used in the research, it was noticed that for the λ_s_ = 50° tool, in a large number of cases, the normality of the distribution was not confirmed, which results from the number of Mann–Whitney U tests performed.
10. The RBF neural network turned out to be a better type of network than the MLP in most cases for modelling the Ra and Rz surface roughness parameters obtained after milling the AZ91D magnesium alloy. The networks obtained for the analysed parameters had from 2 to 10 neurons in the hidden layer.
11. The network obtained as a result of the surface roughness parameter modelling shows satisfactory predictive ability, as evidenced by the obtained values of the R^2^ correlation coefficient for the appropriate training and validation data. For the training data, they are, respectively, Ra = 0.9758 (end face of workpiece), Ra = 0.9947 (lateral face of workpiece), Rz = 0.9604 (end face of workpiece) and Rz = 0.9951 (lateral face of workpiece), and for the validation data, Ra = 0.9773 (end face of workpiece), Ra = 0.9283 (lateral face of workpiece), Rz = 0.9864 (end face of workpiece) and Rz = 0.9804 (lateral face of workpiece). It can, therefore, be concluded that artificial neural networks are an effective tool that can be used to predict surface roughness parameters.
12. The trained networks show the relationships between the input data (v_c_, f_z_, a_p_ and a_e_) and the output data (Ra and Rz parameters), enabling the determination of the appropriate values of the analysed surface roughness parameters after entering the given processing parameters into the network.

In workshop practice, it may be most useful to determine the presented technological parameters of milling, under which machining can take place under conditions defined as safe, effective and efficient. Often, industrial manufacturers do not have the appropriate research equipment, and above all, the time needed to carry out research and the measurement, it is also impossible to withhold or suspend the production in progress. It should be emphasized that in industrial practice, low technological milling parameters are often used, justifying it with safety reasons. Hence, the presented research works may be valuable due to the possibility of implementing the presented scope of technological milling parameters in industrial applications.

In the broadly understood workshop practice, it may be most precious to define such machining parameters for which milling can be finish machining, and constitute a very effective, efficient and safe process (without risk of chip flammability and their ignition). Therefore, the presented research investigations may precious due to the possibility of their application in the industrial and manufacturing practice.

## Figures and Tables

**Table 1:** Comparison of modelling methods using the ANN for the surface roughness parameter.

Machining	Methods	Research Object (Surface Roughness)	Material	Year	Reference
turning	ANN	Ra	AZ61	2018	[29]
turning	ANN-GA	Ra	Ti-6Al-4 V	2015	[26]
milling	ANFIS	Ra	AA6061, AA2024, AA7075	2019	[37]
milling	ANN	Ra	Ti–6Al–4 V	2016	[38]
milling	ANN	Ra	S45C steel	2019	[41]
milling	ANN	Ra	AA6061	2021	[35]
milling	ANN	Ra, Rz, RSm	AZ91D	2021	[30]
milling	ANN	Ra	Ti-6Al-4 V	2019	[39]
milling	ANN	Ra, Rz	Inconel 718	2020	[20]
milling	ANN-GA	Ra	P1.2738	2020	[42]
milling	ANN-GA	Ra	AISI D3	2019	[36]
milling	ANN-GA	Ra	AZ91D	2018	[31]
Low-speed turning	ANN	Ra	AISI316	2015	[33]
dry turning	ANN	Ra, Rz, Rt	AISI 420	2019	[28]
dry milling	ANN	Ra	Co–28Cr–6Mo, Co–20Cr–15W–10Ni	2021	[40]
AWJM	ANN	Ra, Rz, RSm	AZ91D	2018	[32]
AWJM	ANN-GA	Ra	Al 6060	2016	[34]

**Table 2:** Results of the Shapiro–Wilk test for the roughness parameters after milling the AZ91D magnesium alloy, λ_s_ = 20° tool.

	Lateral Face of Workpiece				End Face of Workpiece			
**Comparison**	**v_c_ = 400 m/min**		**v_c_ = 1200 m/min**		**v_c_ = 400 m/min**		**v_c_ = 1200 m/min**	
**Test Result**	**Test**	***p*-Value**	**Test**	***p*-Value**	**Test**	***p*-Value**	**Test**	***p*-Value**
Ra	0.92701	0.5761	0.90335	0.42867	0.86041	0.22971	0.90881	0.46046
Rz	0.92735	0.57844	0.91341	0.48833	0.88383	0.32701	0.92647	0.57249
RSm	0.75007	0.02981	0.87094	0.27027	0.87575	0.29049	0.93743	0.64781
Rsk	0.97334	0.89623	0.99024	0.98054	0.91848	0.52019	0.95693	0.78643
Rku	0.82497	0.12748	0.99407	0.99183	0.89512	0.38352	0.95222	0.75305
**Comparison**	**f_z_ = 0.05 mm/tooth**		**f_z_ = 0.3 mm/tooth**		**f_z_ = 0.05 mm/tooth**		**f_z_ = 0.3 mm/tooth**	
Ra	0.96664	0.85328	0.80452	0.08817	0.94366	0.69193	0.88377	0.32677
Rz	0.95274	0.75678	0.80547	0.08973	0.96051	0.81156	0.969	0.86879
RSm	0.92973	0.59453	0.79941	0.08014	0.87406	0.28324	0.84262	0.17229
Rsk	0.96942	0.87153	0.99326	0.98977	0.9765	0.91507	0.91524	0.49972
Rku	0.84435	0.1773	0.93277	0.6154	0.8805	0.31157	0.74521	0.02686

**Table 3:** Results of the Shapiro–Wilk test for the roughness parameters after milling the AZ91D magnesium alloy, λ_s_ = 50° tool.

	Lateral Face of Workpiece				End Face of Workpiece			
**Comparison**	**v_c_ = 400 m/min**		**v_c_ = 1200 m/min**		**v_c_ = 400 m/min**		**v_c_ = 1200 m/min**	
**Test Result**	**Test**	***p*-Value**	**Test**	***p*-Value**	**Test**	***p*-Value**	**Test**	***p*-Value**
Ra	0.81663	0.10995	0.95541	0.77572	0.91154	0.47691	0.97884	0.92835
Rz	0.7354	0.02168	0.81577	0.10827	0.92973	0.59454	0.919	0.52351
RSm	0.7078	0.01153	0.97664	0.91587	0.92816	0.58389	0.84752	0.18681
Rsk	0.84967	0.19349	0.91019	0.46873	0.95357	0.76262	0.85765	0.21992
Rku	0.6497	0.00264	0.85419	0.20812	0.95542	0.77578	0.9657	0.84702
**Comparison**	**f_z_ = 0.05 mm/tooth**		**f_z_ = 0.3 mm/tooth**		**f_z_ = 0.05 mm/tooth**		**f_z_ = 0.3 mm/tooth**	
Ra	0.65288	0.00288	0.95445	0.74398	0.95581	0.77853	0.7713	0.04632
Rz	0.69372	0.00822	0.94482	0.68391	0.88753	0.34482	0.85179	0.20024
RSm	0.8638	0.24221	0.76603	0.05395	0.71771	0.01454	0.67777	0.00552
Rsk	0.89466	0.38108	0.81947	0.14196	0.95604	0.78018	0.77213	0.04711
Rku	0.98535	0.96103	0.9379	0.64158	0.80611	0.0908	0.86626	0.25161

**Table 4:** Results of the Shapiro–Wilk test for the roughness parameters after milling the AZ31 magnesium alloy, λ_s_ = 20° tool.

	Lateral Face of Workpiece				End Face of Workpiece			
**Comparison**	**v_c_ = 400 m/min**		**v_c_ = 1200 m/min**		**v_c_ = 400 m/min**		**v_c_ = 1200 m/min**	
**Test Result**	**Test**	***p*-Value**	**Test**	***p*-Value**	**Test**	***p*-Value**	**Test**	***p*-Value**
Ra	0.82009	0.11696	0.91865	0.52124	0.98436	0.95652	0.89208	0.36767
Rz	0.96982	0.87409	0.98235	0.94678	0.79289	0.07083	0.9036	0.43009
RSm	0.88425	0.32902	0.95601	0.77995	0.82741	0.13302	0.79527	0.07411
Rsk	0.94807	0.72339	0.84938	0.19257	0.90778	0.45435	0.93953	0.66263
Rku	0.83942	0.16332	0.95506	0.77327	0.92164	0.54058	0.98535	0.96103
**Comparison**	**f_z_ = 0.05 mm/tooth**		**f_z_ = 0.3 mm/tooth**		**f_z_ = 0.05 mm/tooth**		**f_z_ = 0.3 mm/tooth**	
Ra	0.79205	0.0697	0.95492	0.77224	0.76973	0.04487	0.86523	0.24764
Rz	0.87892	0.30443	0.91473	0.49656	0.91012	0.46828	0.82873	0.1361
RSm	0.89477	0.38164	0.98237	0.94689	0.94044	0.66902	0.9143	0.49387
Rsk	0.98474	0.95825	0.82927	0.13738	0.94352	0.69091	0.79541	0.07431
Rku	0.81641	0.10952	0.90784	0.4547	0.91115	0.47455	0.79599	0.07513

**Table 5:** Results of the Shapiro–Wilk test for the roughness parameters after milling the AZ31 magnesium alloy, λ_s_ = 50° tool.

	Lateral Face of Workpiece				End Face of Workpiece			
**Comparison**	**v_c_ = 400 m/min**		**v_c_ = 1200 m/min**		**v_c_ = 400 m/min**		**v_c_ = 1200 m/min**	
**Test Result**	**Test**	***p*-Value**	**Test**	***p*-Value**	**Test**	***p*-Value**	**Test**	***p*-Value**
Ra	0.92022	0.53137	0.98119	0.90899	0.9685	0.86554	0.8087	0.09522
Rz	0.99583	0.99559	0.90767	0.47016	0.93796	0.65154	0.8687	0.26121
RSm	0.93284	0.61589	0.84072	0.1975	0.63891	0.00196	0.62463	0.00131
Rsk	0.87526	0.28839	0.94115	0.66137	0.98373	0.95354	0.64235	0.00216
Rku	0.59038	0.00046	0.77707	0.06705	0.84398	0.17623	0.9798	0.93355
**Comparison**	**f_z_ = 0.05 mm/tooth**		**f_z_ = 0.3 mm/tooth**		**f_z_ = 0.05 mm/tooth**		**f_z_ = 0.3 mm/tooth**	
Ra	0.79241	0.07018	0.83206	0.14414	0.92731	0.57869	0.84128	0.19917
Rz	0.90467	0.43625	0.89212	0.36785	0.92802	0.5828	0.88774	0.37273
RSm	0.97436	0.90245	0.80914	0.096	0.8692	0.29455	0.89009	0.38352
Rsk	0.9049	0.43754	0.94477	0.69982	0.90068	0.43447	0.9524	0.73114
Rku	0.71506	0.01367	0.71978	0.01525	0.82475	0.15451	0.86691	0.28573

**Table 6:** Results of the Mann–Whitney U test for the roughness parameters after milling the magnesium alloy.

	AZ91D				AZ31			
	λ_s_ = 20°		λ_s_ = 50°		λ_s_ = 20°		λ_s_ = 50°	
	Lateral Face	End Face	Lateral Face	End Face	Lateral Face	End Face	Lateral Face	End Face
	*p*-Value	*p*-Value	*p*-Value	*p*-Value	*p*-Value	*p*-Value	*p*-Value	*p*-Value
**v_c_ 400 vs. 1200**								
Ra	-	-	-	-	-	-		
Rz	-	-	0.095238	-	-	-		
RSm	0.015873	-	0.309524	-	-	-		0.055556
Rsk	-	-	-	-	-	-		0.222222
Rku	-	-	0.150794	-	-	-	0.190476	
**f_z_ 0.05 vs. 0.3**								
Ra	-	-	0.031746	0.007937	-	0.007937		
Rz	-	-	0.031746	-	-	-		
RSm	-	-	-	0.007937	-	-		
Rsk	-	-	-	1	-	-		
Rku	-	1	-	-	-	-	0.547619	

**Table 7:** Results of Student’s *t*-test and Cochran’s Q test for the roughness parameters on the lateral face of the AZ91D magnesium alloy, λ_s_ = 20° tool.

λ_s_ = 20° Lateral Face of the AZ91D										
***Student’s t-Test***										
**Roughness Parameter**	**v_c_ 400 vs. 1200**					**f_z_ 0.05 vs. 0.3**				
	**Mean**	**Mean**	**Test**	**df**	***p*-Value**	**Mean**	**Mean**	**Test**	**df**	***p*-Value**
Ra	1.0262	0.4182	5.46025	8	0.000601	0.3352	0.5046	−2.0036	8	0.080066
Rz	4.212	2.348	6.62059	8	0.000166	2.038	2.65	−1.1619	8	0.27873
RSm	-	-	-	-	-	0.206	0.283	−3.0689	8	0.015372
Rsk	-	-	-	-	-	0.0366	0.3976	−1.4108	8	0.195969
Rku	2.3382	4.1638	−4.17206	8	0.003113	4.5826	3.132	1.5866	8	0.151259
***Cochran’s Q test***										
Roughness parameter	**v_c_ 400 vs. 1200**					**f_z_ 0.05 vs. 0.3**				
	Mean	Mean	Test *	df	*p*-Value	Mean	Mean	Test *	df	*p*-Value
Rsk	0.4694	0.1038	0.996435	4.293571	0.37183	-	-	-	-	-

* Test with independent variance estimation.

**Table 8:** Results of Cochran’s Q test for the roughness parameters on the end face of the AZ91D magnesium alloy, λ_s_ = 20° tool.

λ_s_ = 20° End Face of the AZ91D										
*Cochran’s Q Test*										
Roughness Parameter	v_c_ 400 vs. 1200					f_z_ 0.05 vs. 0.3				
	Mean	Mean	Test *	df	*p*-Value	Mean	Mean	Test *	df	*p*-Value
Ra	2.3714	1.3818	3.4085	4.100354	0.026035	0.8712	1.786	-5.9069	4.583435	0.00265
Rz	13.128	6.412	4.3650	4.022686	0.011865	4.166	7.97	-4.6056	4.361437	0.008121
RSm	0.1196	0.1716	−1.0226	4.590582	0.357305	0.1014	0.2248	-3.6712	4.409552	0.018011
Rsk	0.334	−0.3034	3.5067	4.432405	0.020918	0.05	0.2152	-0.7847	4.105744	0.475401
Rku	2.9146	2.2392	1.6676	4.217333	0.167024	-	-	-	-	-

* Test with independent variance estimation.

**Table 9:** Results of Student’s *t*-test and Cochran’s Q test for the roughness parameters on the lateral face of the AZ91D magnesium alloy, λ_s_ = 50° tool.

λ_s_ = 50° Lateral Face of the AZ91D										
***Student’s t-Test***										
**Roughness Parameter**	**v_c_ 400 vs. 1200**					**f_z_ 0.05 vs. 0.3**				
	**Mean**	**Mean**	**Test**	**df**	***p*-Value**	**Mean**	**Mean**	**Test**	**df**	***p*-Value**
**Ra**	0.5052	0.2346	1.929121	8	0.089842	-	-	-	-	-
**RSm**	-	-	-	-	-	0.244	0.251	−0.1441	7	0.889423
**Rsk**	0.3698	0.2158	0.608047	8	0.560018	−0.065	0.2945	−2.2076	7	0.063011
***Cochran’s Q test***										
Roughness parameter	**v_c_ 400 vs. 1200**					**f_z_ 0.05 vs. 0.3**				
	Mean	Mean	Test *	df	*p*-Value	Mean	Mean	Test *	df	*p*-Value
**Rku**	-	-	-	-	-	3.158	3.6792	−0.69778	4.09452	0.52290

* Test with independent variance estimation.

**Table 10:** Results of Student’s *t*-test for the roughness parameters on the end face of the AZ91D magnesium alloy, λ_s_ = 50° tool.

λ_s_ = 50° End Face of the AZ91D										
*Student’s t-Test*										
Roughness Parameter	v_c_ 400 vs. 1200					f_z_ 0.05 vs. 0.3				
	Mean	Mean	Test	df	*p*-Value	Mean	Mean	Test	df	*p*-Value
**Ra**	3.4866	1.3698	6.11039	8	0.000286	-	-	-	-	-
**Rz**	14.148	5.99	10.53708	8	0.000006	4.072	12.896	−8.0618	8	0.000041
**RSm**	0.409	0.1698	15.43258	8	0	-	-	-	-	-
**Rsk**	0.3126	0.359	−0.43022	8	0.678391	-	-	-	-	-
**Rku**	2.2608	2.0778	0.7786	8	0.458621	2.91	2.4866	1.33904	8	0.217355

**Table 11:** Results of Student’s *t*-test and Cochran’s Q test for roughness parameters on the lateral face of the AZ31 magnesium alloy, λ_s_ = 20° tool.

λ_s_ = 20° Lateral Face of the AZ31										
***Student’s t-Test***										
**Roughness Parameter**	**v_c_ 400 vs. 1200**					**f_z_ 0.05 vs. 0.3**				
	**Mean**	**Mean**	**Test**	**df**	***P*-Value**	**Mean**	**Mean**	**Test**	**df**	***p*-Value**
**Ra**	0.865	0.2802	18.87493	8	0	-	-	-	-	-
**Rz**	3.642	1.474	11.44865	8	0.000003	-	-	-	-	-
**RSm**	0.4284	0.228	10.31757	8	0.000007	0.1568	0.2928	-9.9378	8	0.000009
**Rsk**	0.5294	0.1632	1.69294	8	0.128923	0.2238	0.6018	-1.8519	8	0.101177
**Rku**	2.2722	2.8788	−2.46516	8	0.039004	2.9852	3.2926	-0.674	8	0.519308
***Cochran’s Q test***										
Roughness parameter	**v_c_ 400 vs. 1200**					**f_z_ 0.05 vs. 0.3**				
	Mean	Mean	Test *	df	*p*-Value	Mean	Mean	Test *	df	*p*-Value
**Ra**	-	-	-	-	-	0.2492	0.5136	−4.2146	4.955853	0.008535
**Rz**	-	-	-	-	-	1.31	2.542	−5.0265	4.892736	0.004262

* Test with independent variance estimation.

**Table 12:** Results of Student’s *t*-test and Cochran’s Q test for the roughness parameters on the end face of the AZ31 magnesium alloy, λ_s_ = 20° tool.

λ_s_ = 20° End Face of the AZ31										
***Student’s t-Test***										
**Roughness Parameter**	**v_c_ 400 vs. 1200**					**f_z_ 0.05 vs. 0.3**				
	**Mean**	**Mean**	**Test**	**df**	***p*-Value**	**Mean**	**Mean**	**Test**	**df**	***p*-Value**
**Ra**	1.6406	1.2122	4.32639	8	0.002524	-	-	-	-	-
**Rz**	8.828	5.626	6.515186	8	0.000185	-	-	-	-	-
**RSm**	-	-	-	-	-	0.1026	0.1884	−3.8625	8	0.004791
**Rsk**	0.1984	−0.3486	3.274444	8	0.011281	-	-	-	-	-
***Cochran’s Q test***										
Roughness parameter	**v_c_ 400 vs. 1200**					**f_z_ 0.05 vs. 0.3**				
	Mean	Mean	Test *	df	*p*-Value	Mean	Mean	Test *	df	*p*-Value
**Rz**	-	-	-	-	-	4.37	8.092	−4.6895	5.185945	0.004908
**RSm**	0.2312	0.1614	1.229567	4.541987	0.27872	-	-	-	-	-
**Rsk**	-	-	-	-	-	−0.031	0.3898	−1.5343	5.887224	0.176774
**Rku**	2.8686	2.28	1.748142	4.079996	0.153946	2.1782	2.8806	−1.263	4.124669	0.273271

* Test with independent variance estimation.

**Table 13:** Results of Student’s *t*-test and Cochran’s Q test for the roughness parameters on the lateral face of the AZ31 magnesium alloy, λ_s_ = 50° tool.

λ_s_ = 50° Lateral Face of the AZ31										
***Student’s t-Test***										
**Roughness Parameter**	**v_c_ 400 vs. 1200**					**f_z_ 0.05 vs. 0.3**				
	**Mean**	**Mean**	**Test**	**df**	***p*-Value**	**Mean**	**Mean**	**Test**	**df**	***p*-Value**
**Ra**	0.7024	0.32675	4.346335	7	0.00337	-	-	-	-	-
**Rz**	4.01	2.1725	4.419454	7	0.003083	1.116	2.474	−3.3989	8	0.009376
**RSm**	-	-	-	-	-	0.1992	0.2924	−8.8984	8	0.00002
**Rsk**	0.7638	0.3465	1.130161	7	0.295641	0.0098	0.4204	−2.7148	8	0.026458
***Cochran’s Q test***										
Roughness parameter	**v_c_ 400 vs. 1200**					**f_z_ 0.05 vs. 0.3**				
	Mean	Mean	Test *	df	*p*-Value	Mean	Mean	Test *	df	*p*-Value
**Ra**	-	-	-	-	-	0.2106	0.5206	−3.6098	5.986017	0.011279
**RSm**	0.1174	0.14475	−0.34124	4.568971	0.748044	-	-	-	-	-

* Test with independent variance estimation.

**Table 14:** Results of Student’s *t*-test and Cochran’s Q test for the roughness parameters on the end face of the AZ31 magnesium alloy, λ_s_ = 50° tool.

λ_s_ = 50° End Face of the AZ91D					
***Student’s t-Test***					
**f_z_ 0.05 vs. 0.3**					
**Roughness Parameter**	**Mean**	**Mean**	**Test**	**df**	***p*-Value**
**Ra**	0.128	0.263	−2.18913	6	0.071161
**Rz**	0.815	1.2025	−1.29384	6	0.243291
**RSm**	0.037	0.22075	−4.09542	6	0.00639
**Rsk**	−0.0895	0.3225	−1.56914	6	0.167667
**Rku**	4.796	2.54725	3.57914	6	0.011655
***Cochran’s Q test***					
**v_c_ 400 vs. 1200**					
Roughness parameter	Mean	Mean	Test *	df	*p*-Value
**Ra**	2.7644	0.117	8.36535	4.019698	0.001093
**Rz**	13.32	0.708	5.69311	4.014721	0.00465
**Rku**	2.1364	2.4142	−1.16828	5.724052	0.289037

* Test with independent variance estimation.

**Table 15:** Selected networks based on quality (learning, validation) and errors (learning, validation).

Network Name	Quality (Training)	Quality (Validation)	Error (Training)	Error (Validation)	Activation (Hidden)	Activation (Output)
**End face of workpiece**						
**Ra**						
**RBF** 3-9-1	0.975827	0.977331	0.002393	0.009909	Gaussian	Linear
**Rz**						
**MLP** 3-2-1	0.960436	0.986464	0.101807	0.404443	Exponential	Exponential
**Lateral face of workpiece**						
**Ra**						
**RBF** 3-10-1	0.994723	0.948386	0.000176	0.000871	Gaussian	Linear
**Rz**						
**RBF** 3-10-1	0.995174	0.980441	0.001948	0.039403	Gaussian	Linear

**Table 16:** Sensitivity analysis values for the technological parameters: cutting speed v_c_ and feed per tooth f_z_, axial depth of cut a_p_, radial depth of cut a_e_.

Sensitivity Analysis				
**End face of workpiece**		**v_c_**	**f_z_**	**a_p_**
Ra	RBF 3-9-1	11.83949	6.30613	1.43421
Rz	MLP 3-2-1	8.503714	4.79162	1.356
**Lateral face of workpiece**		**v_c_**	**f_z_**	**a_e_**
Ra	RBF 3-10-1	42.32768	3.59427	2.36995
Rz	RBF 3-10-1	18.29399	2.81688	3.5018

## Data Availability

Not applicable.

## Nomenclature

v_c_	cutting speed (m/min)
f_z_	feed per tooth (mm/tooth)
a_p_	axial depth of cut (mm)
a_e_	radial depth of cut (mm)
λ_s_	helix angle (°)
γ	tool rake angle (°)
α	tool clearance angle (°) or significance level (in statistical analysis)
ANN	artificial neural network
MLP	Multi-layered perceptron
RBF	Radial Basis Function
